# Association of SNPs in the TIMP-2 gene and large artery atherosclerotic stroke in southern Chinese Han population

**DOI:** 10.18632/oncotarget.23473

**Published:** 2017-12-18

**Authors:** Tie Guo, Haizhen Hao, Lv Zhou, Feng Zhou, Dan Yu

**Affiliations:** ^1^ Haikou Hospital of Xiangya Medical College of Central South University, Haikou, Hainan 570208, China

**Keywords:** TIMP-2, single-nucleotide polymorphisms, large artery atherosclerotic stroke, southern Chinese Han population

## Abstract

Tissue inhibitor of matrix metalloproteinase 2 (TIMP-2) regulates the extracellular matrix degradation, which involved in vascular remodeling and dysfunction, destabilization of atherosclerotic plaque and many other pathological processes. The rupture of atherosclerotic plaque is the trigger of Large artery atherosclerotic (LAA) stroke. We speculate that the Single nucleotide polymorphisms (SNPs) in TIMP-2 may have an association with LAA stroke. To prove this hypothesis, we conducted this case-control study. 250 LAA stroke patients and 250 healthy controls were collected for the analysis of TIMP-2 polymorphisms. Among six SNPs, we detected no deviation from Hardy-Weinberg equilibrium in control group. There was a significant difference in rs4789936 T allele frequency between patient and control groups (OR = 0.68, 95% CI = 0.51–0.91, *P* = 0.009), which means lower risk of LAA stroke. We observed the rs4789936 had a decreased risk of LAA stroke according to the codominant (OR = 0.64, 95% CI = 0.44–0.92, *P* = 0.026), dominant (OR = 0.62, 95% CI = 0.43-0.88, *P* = 0.008), overdominant (OR = 0.68, 95% CI = 0.48–0.98, *P* = 0.039), log-additive (OR = 0.68, 95% CI = 0.51–0.91, *P* = 0.009) models analyses. However, these findings could only validate under dominant model (OR = 0.65, 95% CI = 0.42–1.00, *P* = 0.049) after adjustment of gender and age. The results indicate a potential association between TIMP-2 variants and LAA stroke risk in southern Chinese Han population.

## INTRODUCTION

Ischemic stroke, one of the most complex vascular disease with highly morbidity and mortality, continues to be a major global health problem among worldwide [1]. Conventional risk factors like hypertension, dyslipidemia, drinking and smoking are relation to the etiology of ischemic stroke [2]. Furthermore, genetic factors are considered to play an important role in the susceptibility to ischemic stroke [3]. Understanding the genetic polymorphisms is an essential part for evolving new therapeutic strategies and selecting molecular markers that may help identify people at high risk [4].

The LAA stroke is a subtype of TOAST (Trial of Org 10172 in Acute Stroke Treatment) classification [5]. LAA stroke has the highest risk of early neurological deterioration and early stroke recurrence than other acute ischemic stroke (AIS) subtypes [6]. The process of LAA stroke is usually begin with the vulnerable plaque rupture, accompanying by the formation of thrombus and occlusion of a cerebral artery, and leading to irreversible brain damage, blood brain barrier (BBB) disruption, and cellular edema [7]. The rupture of vulnerable atherosclerotic plaques is an important cause of cerebral infarction [8]. The fibrous cap thickness of atherosclerotic plaque is an important index to determine its risk, the minimal fibro cap thickness >101 μm often means low risk, while the minimal fibro cap thickness <65 μm was recognized as a high risk index [9]. The rupture of vulnerable plaques is related to the activity of Matrix metalloproteinases (MMPs) [10]. Whether cerebral infarction occurs in atherosclerotic patients, is related to the levels of MMPs and TIMPs in blood [11].

MMPs are a family of zinc-binding proteinases that play an important role in degrading the extracellular matrix (ECM) and pathological remodeling of blood vessels [12]. Matrix metalloproteinase-2 (MMP-2), one of important members of the MMPs family, has several roles in cerebral vascular diseases. The imbalance of MMP-2 and TIMP-2 plays a very important role in the formation of arterial aneurysm [13]. The atherosclerotic plaques have a high expression of MMP-2, and the unstable plaques have higher MMP-2 expression than the stable plaques [14]. Genetical studies demonstrated that MMP-2 polymorphisms have been found to be associated with the development of lacunar stroke [15]. Additionally, SNPs in MMP-2 gene was correlated with the functional outcome after stroke [16].

Conventionally, we believed that TIMP-2 is an important natural endogenous inhibitor of MMP-2. In fact, the relationship between TIMP-2 and MMP-2 is not simply as an inhibitor and substrate. There is a concentration dependent relationship between the TIMP-2 and MMP-2. When the concentration of TIMP-2 is low, its carboxyl-terminal domains and membrane type-1 matrix metalloproteinase (MT1-MMP/MMP-14), and carboxyl-terminal hemopexin-like domain (PEX) of MMP-2, can form a complex, which activate the pro-MMP-2, promote its maturity; when the concentration of TIMP-2 is high, TIMP-2 can combine with MMP-2 by its N-terminal domain, inhibiting MMP-2 activity. The relationship between TIMP-2 and MMP-2 is unique in the MMPs family, and many physiological and pathological processes are dependent on the balance between MMP-2 and TIMP-2 [17–18]. In cerebrovascular diseases, the imbalance of MMP-2 and TIMP-2 can cause vascular remodeling and dysfunction [19], cerebral aneurysm formation [20], disruption of blood brain barrier [21], destabilization of atherosclerotic plaque [22], hemorrhage transformation after cerebral ischemia [23]. Knockout of TIMP-1 and gene transfer of TIMP-1 and TIMP-2 in mice can accelerate the atherosclerosis formation and make atherosclerotic plaque unstable [24].

During the past years different SNPs within the MMP-2 gene and their association with cerebrovascular diseases were studied. However, data is lacking regarding the role of TIMP-2 polymorphisms in the susceptibility to ischemic stroke. Therefore, in the present investigation, we aimed to determine whether relevant SNPs within the TIMP-2 gene are associated with the susceptibility of LAA stroke in southern Chinese Han population. We carefully searched the databases of 1000 Genomes Project (http://www.1000genomes.org/) and dbSNP (https://www.ncbi.nlm.nih.gov/projects/SNP/), choose the SNPs which had minor allele frequencies (MAFs) >5% in Chinese Han Beijing (CHB) population, and had some previous studies. Finally, A total of six SNPs (i.e., rs2277698, rs2009196, rs7342880, rs11654470, rs2003241, rs4789936) were selected for further genotyping.

## RESULTS

A total of 250 unrelated LAA stroke patients (167 female and 83 male; mean age = 64.12 ± 10.98 years) and 250 unrelated healthy controls (152 female and 98 male; mean age = 48.30 ± 12.31 years) involved in this case-control study were presented in Table 1. There were no significant differences in age (*P* = 0.063) and gender (*P* = 0.193) observed between the case and control groups.

**Table 1:** Distributions of age and gender in large artery atherosclerotic stroke patients and controls

Variable	Case (*n* = 250)	Control (*n* = 250)	*P*
Gender (*n*, %)			0.193
male	83 (33.2%)	98 (39.2%)	
female	167 (66.8%)	152 (60.8%)	
Age (year)	64.12 ± 10.98	48.30 ± 12.31	0.063

*P* < 0.05 indicates statistical significance.

Basic information and allele frequencies of rs2277698, rs2009196, rs7342880, rs11654470, rs2003241, and rs4789936 in LAA stroke patients and healthy controls were displayed in Table 2. It is obvious that information obtained from the table with regard to the SNPs and their chromosomal position, allele, minor allele frequency for cases and controls, and HWE test results. None of the SNPs deviated from the Hardy-Weinberg equilibrium in control group (*P >* 0.05). The Minor allele frequency in the rs4789936 was 0.227 for cases compared with 0.300 for controls, which had a significant difference in allele frequency between cases and control groups (OR = 0.68, 95% CI = 0.51–0.91, *P* = 0.009). In addition, the C allele frequency distribution of SNP rs2003241 existed a marginal significant difference between patients with LAA stroke and control subjects (OR = 0.75, 95% CI = 0.56–1.00, *P* = 0.050).

**Table 2:** Basic characteristics and allele frequencies of the six SNPs

SNP	Genes	Chr	Position	Allele	Minor allele frequency		HWE *P* value	OR (95%CI)	*P^a^*
					Case	Control			
rs2277698	TIMP2	17q25.3	76867017	C>T	0.247	0.237	0.7265	1.06 (0.79–1.41)	0.711
rs2009196	TIMP2	17q25.3	76870581	G>C	0.508	0.458	0.7013	1.22 (0.95–1.57)	0.112
rs7342880	TIMP2	17q25.3	76874512	C>A	0.177	0.215	0.4525	0.78 (0.57–1.07)	0.129
rs11654470	TIMP2	17q25.3	76877331	T>C	0.327	0.333	0.8865	0.98 (0.75–1.27)	0.858
rs2003241	TIMP2	17q25.3	76885117	T>C	0.218	0.271	0.1096	0.75 (0.56–1.00)	0.050
rs4789936	TIMP2	17q25.3	76897974	C>T	0.227	0.300	0.7632	0.68 (0.51–0.91)	0.009^*^

SNP: single nucleotide polymorphism; OR = odds ratio; 95% CI = 95% confidence interval;

HWE: Hardy-Weinberg equilibrium;

^*^P < 0.05 indicates statistical significance;

^a^*P* values were calculated using two-sided Chi-squared test/Fisher's exact test.

Comparisons of the SNP genotypes and the risk of LAA stroke under the genetic models were presented in Table 3. We observed a decreased risk of LAA stroke in rs4789936 according to the codominant model(OR = 0.64, 95% CI = 0.44–0.92, *P* = 0.026 for the C/T genotype), dominant model (OR = 0.62, 95% CI = 0.43-0.88, *P* = 0.008), overdominant model (OR = 0.68, 95% CI = 0.48–0.98, *P* = 0.039), log-additive model(OR = 0.68, 95% CI = 0.51–0.91, *P* = 0.009) analyses. However, these findings could only validate by analysis of dominant models (OR = 0.65; 95% CI = 0.42–1.00, *P* = 0.049) after adjustment of gender and age. In addition, we also observed a statistically significant association between the decreased risk of LAA stroke and rs2003241 under codominant model (OR = 0.63, 95% CI = 0.43–0.91, *P* = 0.045 for the T/C genotype), dominant model (OR = 0.65, 95% CI = 0.45–0.92, *P* = 0.016) and overdominant model (OR = 0.64, 95% CI = 0.44–0.92, *P* = 0.015) without adjustment of gender and age.

**Table 3:** *TIMP2* SNPs genotypes and the risk of large artery atherosclerotic stroke

SNP	Genotype	Control	Case	Adjustment analysis		Crude analysis	
		*N* (%)	*N* (%)	OR (95% CI)^a^	*P^a^*	OR (95% CI)^b^	*P^b^*
Rs2277698							
	C/C	146 (58.6%)	140 (56.2%)	1.00		1.00	
Codominant	C/T	88 (35.3%)	95 (38.1%)	1.03 (0.66–1.63)	0.990	1.13 (0.78–1.63)	0.810
	T/T	15 (6.0%)	14 (5.6%)	1.04 (0.43–2.50)		0.97 (0.45–2.09)	
	C/C	146 (58.6%)	140 (56.2%)	1.00		1.00	
Dominant	C/T+T/T	103 (41.4%)	109 (43.8%)	1.04 (0.67–1.60)	0.880	1.10 (0.77–1.57)	0.590
	C/C+C/T	234 (94.0%)	235 (94.1%)	1.00		1.00	
Recessive	T/T	15 (6.0%)	14 (5.6%)	1.02 (0.43–2.43)	0.960	0.93 (0.44–1.97)	0.850
	C/C+T/T	161 (64.7%)	154 (61.9%)	1.00		1.00	
Overdominant	C/T	88 (35.3%)	95 (38.1%)	1.03 (0.66–1.61)	0.890	1.13 (0.78–1.63)	0.520
Log-additive	—	—	—	1.03 (0.73–1.45)	0.880	1.06 (0.79–1.41)	0.710
Rs2009196							
	C/C	71 (28.6%)	66 (26.4%)	1.00		1.00	
Codominant	C/G	127 (51.2%)	114 (45.6%)	0.75 (0.45–1.26)	0.200	0.97 (0.63–1.47)	0.210
	G/G	50 (20.2%)	70 (28.0%)	1.19 (0.65–2.18)		1.51 (0.92–2.47)	
Dominant	C/C	71 (28.6%)	66 (26.4%)	1.00		1.00	
	C/G+G/G	177 (71.4%)	184 (73.6%)	0.88 (0.54–1.42)	0.600	1.12 (0.75–1.66)	0.580
	C/C+C/G	198 (79.8%)	180 (72.0%)	1.00		1.00	
Recessive	G/G	50 (20.2%)	70 (28.0%)	1.43 (0.87–2.37)	0.160	1.54 (1.02–2.33)	0.040
	C/C+G/G	121 (48.8%)	136 (54.4%)	1.00		1.00	
Overdominant	C/G	127 (51.2%)	114 (45.6%)	0.69 (0.45–1.06)	0.092	0.80 (0.56–1.14)	0.210
Log-additive	—	—	—	1.08 (0.80–1.46)	0.600	1.22 (0.95–1.56)	0.120
Rs7342880							
	C/C	151 (60.6%)	164 (65.9%)	1.00		1.00	
Codominant	A/C	89 (35.7%)	82 (32.9%)	1.12 (0.71–1.76)	0.330	0.85 (0.58–1.23)	0.140
	A/A	9 (3.6%)	3 (1.2%)	0.34 (0.07–1.74)		0.31 (0.08–1.15)	
Dominant	C/C	151 (60.6%)	164 (65.9%)	1.00		1.00	
	A/C+A/A	98 (39.4%)	85 (34.1%)	1.05 (0.67–1.63)	0.840	0.80 (0.55–1.15)	0.230
Recessive	C/C+A/C	240 (96.4%)	246 (98.8%)	1.00		1.00	
	A/A	9 (3.6%)	3 (1.2%)	0.33 (0.06–1.66)	0.160	0.33 (0.09–1.22)	0.073
Overdominant	C/C+A/A	160 (64.3%)	167 (67.1%)	1.00		1.00	
	A/C	89 (35.7%)	82 (32.9%)	1.16 (0.74–1.82)	0.530	0.88 (0.61–1.28)	0.510
Log-additive	—	—	—	0.96 (0.64–1.43)	0.840	0.77 (0.55–1.06)	0.110
rs11654470							
	T/T	111 (44.8%)	108 (43.4%)	1.00		1.00	
Codominant	T/C	109 (44.0%)	119 (47.8%)	0.80 (0.51–1.26)	0.630	1.12 (0.77–1.63)	0.550
	C/C	28 (11.3%)	22 (8.8%)	0.97 (0.36–2.59)		0.81 (0.44–1.50)	
Dominant	T/T	111 (44.8%)	108 (43.4%)	1.00		1.00	
	T/C+C/C	137 (55.2%)	141 (56.6%)	0.82 (0.53–1.27)	0.370	1.06 (0.74–1.51)	0.760
Recessive	T/T+T/C	220 (88.7%)	227 (91.2%)	1.00		1.00	
	C/C	28 (11.3%)	22 (8.8%)	1.06 (0.40–2.79)	0.900	0.76 (0.42–1.37)	0.360
Overdominant	T/T+C/C	139 (56.0%)	130 (52.2%)	1.00		1.00	
	T/C	109 (44.0%)	119 (47.8%)	0.80 (0.52–1.25)	0.330	1.17 (0.82–1.66)	0.390
Log-additive	—	—	—	0.88 (0.61–1.26)	0.490	0.98 (0.74–1.28)	0.850
Rs2003241							
	T/T	126 (51.0%)	153 (61.7%)	1.00		1.00	
Codominant	T/C	108 (43.7%)	82 (33.1%)	0.80 (0.51–1.26)	0.630	0.63 (0.43–0.91)	0.045^*^
	C/C	13 (5.3%)	13 (5.2%)	0.97 (0.36–2.59)		0.82 (0.37–1.84)	
Dominant	T/T	126 (51.0%)	153 (61.7%)	1.00		1.00	
	T/C+C/C	121 (49.0%)	95 (38.3%)	0.82 (0.53–1.27)	0.370	0.65 (0.45–0.92)	0.016^*^
Recessive	T/T+T/C	234 (94.7%)	235 (94.8%)	1.00		1.00	
	C/C	13 (5.3%)	13 (5.2%)	1.06 (0.40–2.79)	0.900	1.00 (0.45–2.19)	0.990
Overdominant	T/T+C/C	139 (56.3%)	166 (66.9%)	1.00		1.00	
	T/C	108 (43.7%)	82 (33.1%)	0.80 (0.52–1.25)	0.330	0.64 (0.44–0.92)	0.015^*^
Log-additive	—	—	—	0.88 (0.61–1.26)	0.490	0.74 (0.55–1.00)	0.045
Rs4789936							
	C/C	120 (48.4%)	150 (60.2%)	1.00		1.00	
Codominant	C/T	107 (43.1%)	85 (34.1%)	0.65 (0.42–1.03)	0.140	0.64 (0.44–0.92)	0.026^*^
	T/T	21 (8.5%)	14 (5.6%)	0.62 (0.25–1.52)		0.53 (0.26–1.09)	
Dominant	C/C	120 (48.4%)	150 (60.2%)	1.00		1.00	
	C/T+T/T	128 (51.6%)	99 (39.8%)	0.65 (0.42–1.00)	0.049^*^	0.62 (0.43–0.88)	0.008^*^
Recessive	C/C+C/T	227 (91.5%)	235 (94.4%)	1.00		1.00	
	T/T	21 (8.5%)	14 (5.6%)	0.74 (0.31–1.77)	0.500	0.64 (0.32–1.30)	0.210
Overdominant	C/C+T/T	141 (56.9%)	164 (65.9%)	1.00		1.00	
	C/T	107 (43.1%)	85 (34.1%)	0.69 (0.44–1.07)	0.096	0.68 (0.48–0.98)	0.039^*^
Log-additive	—	—	—	0.72 (0.51–1.02)	0.063	0.68 (0.51–0.91)	0.009^*^

SNPs: Single nucleotide polymorphisms; OR: odds ratio; 95% CI = 95% confidence interval.

^*^P < 0.05 indicates statistical significance;

^a^*P* values were calculated by unconditional logistic regression adjusted for gender and age;

^b^*P* values were calculated by unconditional logistic regression without adjustment of gender and age.

**Figure 1:**
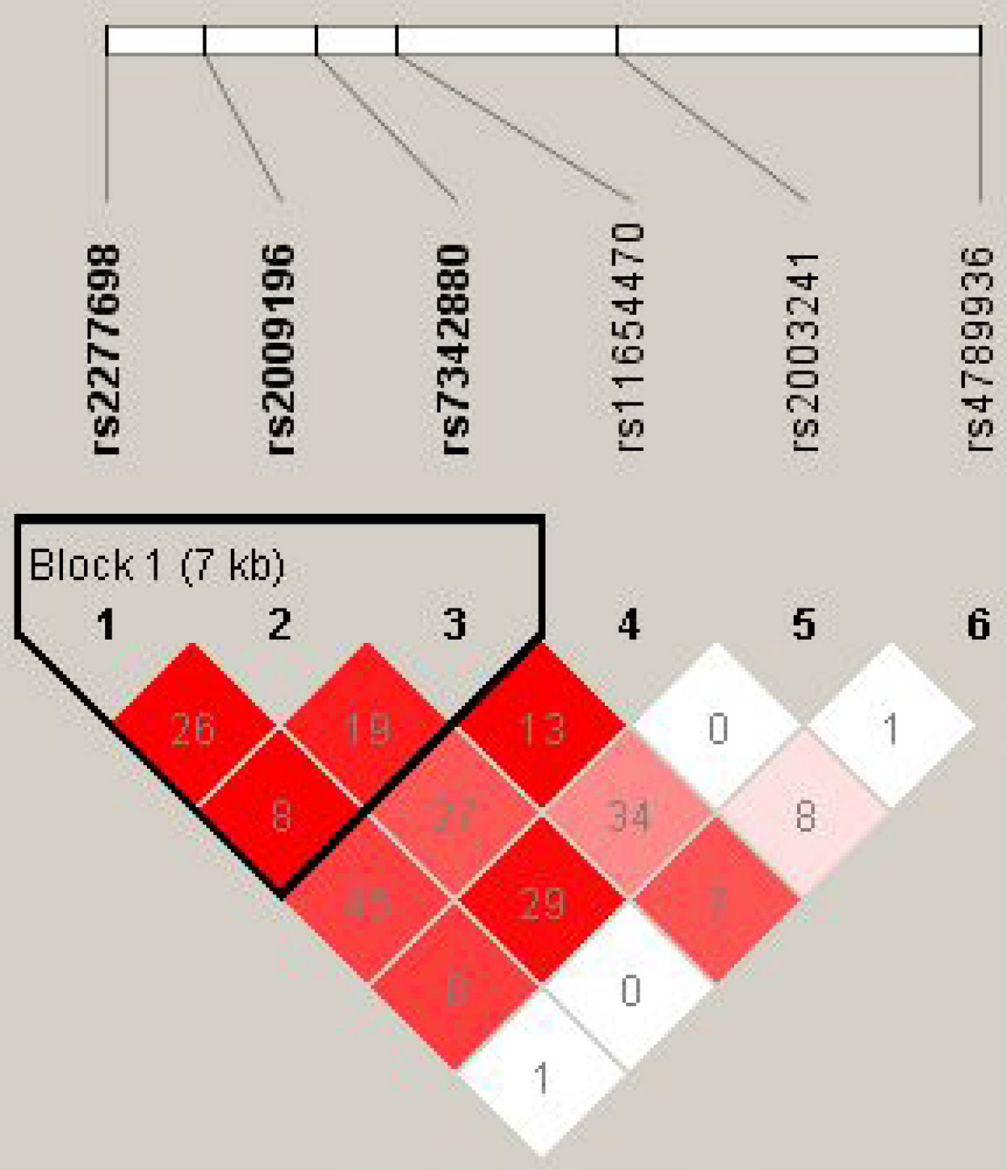
Haplotype block map for the 6 SNPs in the TIMP2 gene in our experiment Red squares indicate statistically significant associations between a pair of SNPs, as measured by D’; darker shades of red indicate higher D’ values.

The results for the association between the TIMP-2 haplotype and the risk of LAA stroke are shown in Table 4 and Figure 1. In the linkage analyses, three TIMP-2 SNPs (rs2277698, rs2009196 and rs7342880) mapped in a 7kb LD block, however, there were no significant differences in haplotype frequencies among any of the groups.

**Table 4:** *TIMP2* haplotype frequencies and the association with large artery atherosclerotic stroke

rs2277698	rs2009196	rs7342880	Freq	Adjusted Analysis		Crude Analysis	
				OR (95% CI)^a^	*P*^a^	OR (95% CI)^b^	*P*^b^
C	G	C	0.4732	1.00	—	1.00	—
T	C	C	0.2407	0.99 (0.68–1.42)	0.940	0.95 (0.70–1.29)	0.750
C	C	A	0.1843	0.90 (0.58–1.38)	0.620	0.70 (0.49–1.00)	0.052
C	C	C	0.0907	0.76 (0.43–1.34)	0.350	0.71 (0.44–1.12)	0.140

OR = odds ratio; 95% CI = 95% confidence interval;

^a^*P* values were calculated by unconditional logistic regression adjusted for age and gender;

^b^*P* values were calculated by unconditional logistic regression without adjusted by age and gender.

## DISCUSSION

We conducted this case-control study to investigate the relationship between TIMP-2 polymorphisms and LAA stroke in a southern Chinese Han population. We found that the rs4789936 polymorphism in TIMP-2 gene was associated with the lower risk of LAA stroke, and rs2003241 polymorphism only had a marginal association with the lower risk of LAA stroke. However, we didn't observe any associations between the polymorphisms rs2277698, rs2009196, rs7342880, rs11654470 and the LAA stroke. And no significant difference was found under haplotype analysis after adjustment of gender and age.

The SNPs rs2003241 and rs11654470 were studied for the susceptibility and survival of breast cancer [25], rs2009196 was studied in the abdominal aortic aneurysm [26], but no positive results were found. Rs2277698 is a SNP of many studies, which involved in pleural thickening calcification [27], abnormal sperm morphology [28], preterm prelabor rupture of membranes [29] and other pathological processes. The combination of rs7342880 and other loci can well diagnose the progression of rheumatoid arthritis of the knee [30]. However, in the present study, only rs2003241 had a marginal association with LAA stroke, and no significant relationship with LAA stroke was found in other loci.

The SNP rs4789936 localized in the intron of the gene, has been proved to be related to the knee osteoarthritis risk in Chinese Han population [31]. To the best of our knowledge, this is the first study to confirm the association between genetic variatins rs4789936 of TIMP-2 and the risk of LAA stroke in southern Chinese Han population.

Single nucleotide polymorphisms that cause protein structural defects or affect TIMP-2 transcription efficiency can cause cerebral infarction or cerebral hemorrhage by affecting the balance between TIMP-2 and MMP-2 [32]. Previous study has shown that the specificity of TIMP-2 for MMP-2 binding and inhibition can be modified by single amino acid mutations [33]. MMP-2 and TIMP-2 expression can be regulated by fluid shear stress, which are important factors contribute to atherosclerosis [34]. A majority of LAA stroke events are associated with the rupture of atherosclerotic plaque, MMP-2 activity plays a pivotal role in that process [35]. TIMP-2 is the most important endogenous regulator of MMP-2. Data from an *in vitro* stroke model of a human brain microvascular endothelial cell line show favorable effect of statin pretreatment on the MMPs metabolism, mainly by induced the expression and secretion of TIMP-1 and TIMP-2 [36]. In an animal model, the overexpression of TIMP-2 had shown the effect of inhibit atherosclerotic plaque development and destabilization [37].

The T allele of rs4789936 has a protective effect on LAA stroke. We speculate, rs4789936 itself may affect the transcription rate of TIMP-2 or in linkage disequilibrium with other polymorphisms. Ultimately, through the function of TIMP2 itself or by adjusting the balance of TIMP2/MMP2, it plays a protective role in the pathological process such as ameliorates vascular dysfunction and remodeling caused by hypertension [19], inhibits neointimal hyperplasia [38], carotid artery stenosis [39], rupture of atherosclerotic plaques [22], and reduces the incidence of LAA stroke. But these speculations were needed to further investigate.

The results presented here suffer from some limitations. Some other genetic polymorphisms may play a role in the development of LAA stroke, but our study only investigated the association between part of the TIMP-2 SNPs and the risk of LAA stroke. In addition, the sample size is relatively small, which may limit the statistical power to find the differences between groups.

Taken together, we analyzed the intron region of TIMP-2 and parts of its coding exon region. 6 SNPs were selected, and there were no published studies regarding the association between these SNPs and the risk of LAA stroke in southern Chinese Han population. We proved that, after adjusted by gender and age, the SNP rs4789936 T allele and C/T+T/T under the dominant model were associated with the decreased risk of LAA stroke. In addition, we also observed a statistically significant association between the risk of LAA stroke and the distribution of genotypes of SNP rs2003241 by logistic regression analysis without adjustment of gender and age. Although this study might provide new insights to understand the association of TIMP-2 gene with LAA stroke, and contribute to the early detection of LAA stroke, these results await further confirmation by larger sample size studies.

## MATERIALS AND METHODS

### Study participants

A total of 250 unrelated LAA stroke patients and 250 healthy controls in a large cohort of southern Chinese Han population during 2016-2017 were recruited for this case-control study. The cases were recently diagnosed with LAA stroke for the first time, without any other severe diseases, treated at the Xiangya Haikou Hospital. LAA stroke was diagnosed according to TOAST (Trial of Org 10172 in Acute Stroke Treatment) criteria [5]. Control subjects without any clinical signs or medical history of any types of stroke, and without any other severe diseases, were selected from the health checkup center of the Xiangya Haikou Hospital. Blood samples were collected respectively at the time of initial diagnose, after giving an informed consent from all the participants. This study protocol was approved by the Human Research Ethics Committee of Xiangya Haikou Hospital.

### SNP selection and genotyping

Candidate SNPs in the TIMP-2 gene from the databases of 1000 Genomes Project (http://www.1000genomes.org/) and dbSNP (https://www.ncbi.nlm.nih.gov/projects/SNP/) with MAFs >5% in Chinese Han Beijing (CHB) population, and had some previous studies were chosen. A total of six SNPs (i.e., rs2277698, rs2009196, rs7342880, rs11654470, rs2003241, rs4789936) were selected for further genotyping. These SNPs were analyzed in vascular diseases or other diseases, such as the breast cancer [25], abdominal aortic aneurysm [26], knee osteoarthritis [31]. Genomic DNA was extracted from peripheral blood of cases and controls using the GoldMag whole blood genomic DNA purification kit (GoldMag Co. Ltd., Xi’an, China), as recommended by the manufacturer's instructions [40]. DNA concentration was determined by the NanoDrop 2000C spectrophotometer (Thermo Scientific, Waltham, MA, USA). Sequenom MassARRAY Assay Design 3.0 software (San Diego, CA, USA) was used to design primers for amplification process and single base extension reactions [41]. SNP genotyping was carried out by Sequenom MassARRAY RS1000 (Sequenom, SanDiego, CA). Sequenom Typer 4.0 software was used to manage and analyze SNP genotypic data.

### Statistical analysis

Statistical analyses were carried out using Microsoft Excel and SPSS 17.0 (SPSS, Chicago, IL, USA) software. Deviation from Hardy-Weinberg equilibrium (HWE) was tested for control subjects to measure the distribution of the polymorphism using a Chi-square test [42]. The Haploview software package (version 4.2) and SHEsis software platform (http://analysis.bio-x.cn/myanalysis.php) were used for analyses of linkage disequilibrium, haplotype construction [43, 44]. Associations between haplotypes and LAA stroke risk were analyzed by SNPStats (http://bioinfo.iconcologia.net/SNPstats) [45]. We used the unconditional logistic regression analysis adjusted by age and gender to determine the association between the haplotyes and LAA stroke. Two-sided *P*-value less than 0.05 was considered statistically significant. The risk associated with individual genotypes and allele was calculated as the odds ratios (OR) with their 95% confidence interval (95% CI) based on logistic regression models analysis.
